# The Contribution of Mathematical Modeling to Understanding Dynamic Aspects of Rumen Metabolism

**DOI:** 10.3389/fmicb.2016.01820

**Published:** 2016-11-23

**Authors:** André Bannink, Henk J. van Lingen, Jennifer L. Ellis, James France, Jan Dijkstra

**Affiliations:** ^1^Animal Nutrition, Wageningen Livestock Research, Wageningen University and ResearchWageningen, Netherlands; ^2^Animal Nutrition Group, Wageningen University and ResearchWageningen, Netherlands; ^3^Centre for Nutrition Modelling, Department of Animal Biosciences, University of Guelph, GuelphON, Canada

**Keywords:** dynamic models, rumen digestion, rumen microbiota, volatile fatty acids, rumen regulatory mechanisms

## Abstract

All mechanistic rumen models cover the main drivers of variation in rumen function, which are feed intake, the differences between feedstuffs and feeds in their intrinsic rumen degradation characteristics, and fractional outflow rate of fluid and particulate matter. Dynamic modeling approaches are best suited to the prediction of more nuanced responses in rumen metabolism, and represent the dynamics of the interactions between substrates and micro-organisms and inter-microbial interactions. The concepts of dynamics are discussed for the case of rumen starch digestion as influenced by starch intake rate and frequency of feed intake, and for the case of fermentation of fiber in the large intestine. Adding representations of new functional classes of micro-organisms (i.e., with new characteristics from the perspective of whole rumen function) in rumen models only delivers new insights if complemented by the dynamics of their interactions with other functional classes. Rumen fermentation conditions have to be represented due to their profound impact on the dynamics of substrate degradation and microbial metabolism. Although the importance of rumen pH is generally acknowledged, more emphasis is needed on predicting its variation as well as variation in the processes that underlie rumen fluid dynamics. The rumen wall has an important role in adapting to rapid changes in the rumen environment, clearing of volatile fatty acids (VFA), and maintaining rumen pH within limits. Dynamics of rumen wall epithelia and their role in VFA absorption needs to be better represented in models that aim to predict rumen responses across nutritional or physiological states. For a detailed prediction of rumen N balance there is merit in a dynamic modeling approach compared to the static approaches adopted in current protein evaluation systems. Improvement is needed on previous attempts to predict rumen VFA profiles, and this should be pursued by introducing factors that relate more to microbial metabolism. For rumen model construction, data on rumen microbiomes are preferably coupled with knowledge consolidated in rumen models instead of relying on correlations with rather general aspects of treatment or animal. This helps to prevent the disregard of basic principles and underlying mechanisms of whole rumen function.

## Introduction

A large part of human edible protein is produced from human inedible resources by ruminants. Ruminant production systems can range from extensive pasture-based systems with low external inputs, to intensive production systems with high external inputs and intensive farm management. Invariably, rumen function is key to ruminant performance, to the level of production intensity and efficiency of nutrient use achieved, and to the level and type of emissions into the environment. A quantitative understanding of rumen function is a prerequisite of ‘engineering’ rumen function and, given its boundaries, to predict how to make best use of available resources.

The present paper delivers an overview of how mathematical modeling aids in understanding the variation in whole rumen function and addresses several concepts concerning the dynamics of rumen fermentation. It describes how a quantitative integration of the sources of information that have become available from rumen research (varying from intra-microbial processes to that of the ruminant host) can be achieved, and what further modeling efforts are foreseen. It is postulated that there is merit in adopting a dynamic rumen modeling approach to understand and predict the more dynamic aspects of rumen microbial metabolism and rumen fermentation conditions. This holds for concepts generally used in current rumen models. First, representing the effect of feeding frequency on rumen metabolism requires the dynamics of rumen microorganism and feed substrate degradation to be accounted for. Second, next to the importance of solids turnover for rumen function, there can also be a profound impact of fluid turnover on microbial metabolism in the gastrointestinal fermentation compartments, requiring fluid dynamics to be taken into account. Third, the impact of the dynamics of the nitrogen exchange between the host and the rumen fermentation compartment needs to be accounted for to predict when rumen nitrogen levels are approached that start to limit microbial protein synthesis. Fourth, predicting of end-products of fermentation requires not only the dynamics of microbial growth to be taken into account, but also the impact of rumen fermentation conditions on production of volatile fatty acids (VFA; and hydrogen and methane). Fifth, adaptive processes in the rumen wall have an important role when quantifying response to dietary changes and are to be accounted for.

### Dynamics of Substrate Degradation and Microbial Growth

**FIGURE 1:**
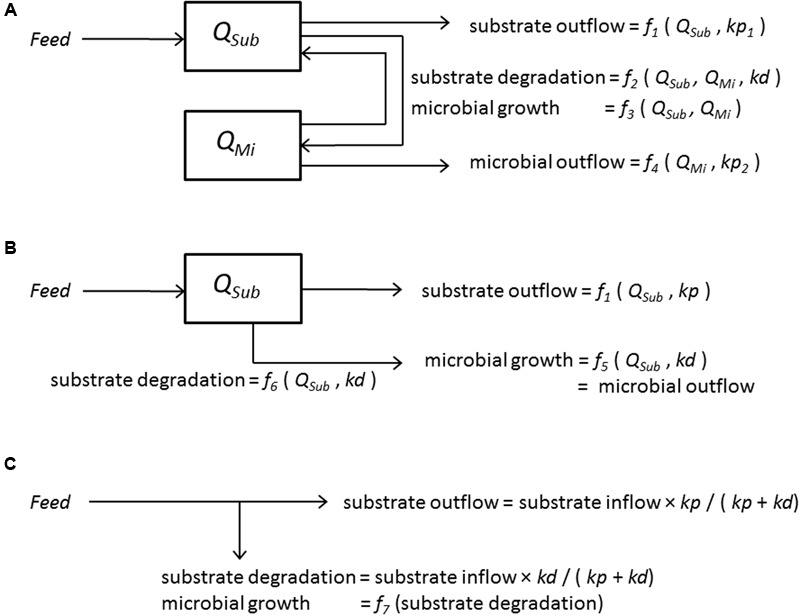
**Modeling approaches to quantify microbial activity and substrate degradation in the rumen (adapted from Bannink et al., 2006a).** Boxes indicate state variables of rumen substrate (*Sub*) (*Q_Sub_*, quantity of rumen substrate, g or mol); rumen micro-organisms (*Mi*) (*Q_Mi_*, quantity of rumen micro-organisms, g) determined by inflows and outflows indicated by arrows (g/d or mol/d) which are defined by feed intake (*Feed*, g/d), fractional rate of substrate degradation (*kd*, /d) and fractional rate of rumen passage (*kp*, /d). **(A)** Representation of a dynamic model including interrelationships between substrate and micro-organisms. **(B)** Representation of a dynamic model without interrelationships between substrate and micro-organisms. A dynamic model that represents all in- and out-flows by mass action forms (using *kd* and *kp*) can be simplified into a static model (depicted in **C**). **(C)** Representation of a static model.

Microbial fermentation in the rumen is generally simplified by assuming that the rumen is a chemostat with ideal mixing of contents and continuous inflow of feed substrate and subsequent outflow of rumen contents. Therefore, the basic approach to representing rumen microbial activity in mathematical terms corresponds to that used for chemostat cultures (Dijkstra et al., 1998). The quantity of substrate (to be utilized by micro-organisms) and the mass of micro-organisms present in the rumen are variables in more complex rumen models. In mechanistic rumen models, these quantities are calculated by the use of differential equations that describe the dynamics of interactions between the processes of inflow of feed substrate, microbial substrate degradation, microbial growth, and outflow of undigested feed and microbial matter. Some processes can be assumed to follow mass action kinetics but many are, in essence, of a non-linear nature, and this requires complex models comprising non-linear differential equations to be developed (**Figure 1A**).

Despite their typically non-linear nature, representation of these processes is often simplified in rumen models. Usually, in simplified rumen models, mass action kinetics are assumed. This allows the non-linear differential equations to become linear without, however, any interaction between the micro-organisms and substrates (**Figure 1B**). In which case, the formulation reduces into that of a static model (excluding the need to represent rumen concentrations and time as model variables). The outflow rates of substrate and micro-organisms can then basically be calculated directly from ratios of the fractional rate of substrate degradation and rumen outflow (**Figure 1C**). With some exceptions, the latter is the basic approach adopted in most models and protein evaluation systems for ruminants applied in practice (e.g., Danfaer et al., 2006; Tylutki et al., 2008; Van Duinkerken et al., 2011; Volden, 2011). These static rumen models are useful as they enable key nutritional characteristics required for diet optimization to be calculated. Such characteristics include the amount of microbial protein synthesized, rumen bypass protein and rumen N availability of different feedstuffs. However, from a mathematical as well as a microbiological viewpoint (i.e., the rumen following the principles of chemostat theory), these static models cannot predict the same accuracy of response to dietary changes as non-linear dynamic models.

Non-linear dynamic representations are used to explain more complex aspects of observed variation in feed substrate degradation and microbial growth. Variation in observed degradation cannot be fully explained without representing the variation in the intrinsic physical and chemical characteristics of feed substrates. Therefore, Dijkstra et al. (1992) adopted the concept of using *in situ* degradation characteristics derived under standardized conditions in the rumen to generate model inputs, which are the same inputs as those used in static rumen models. The earlier dynamic rumen model of Baldwin et al. (1987) initially adopted a different approach using fixed intrinsic degradation characteristics of fiber for specific forage classes (grasses, legumes, maize), of starch, and of degradable protein, leaving all observed variation in substrate degradation to be explained by the dynamic model through a combined representation of microbial activity and particle dynamics (particle size distribution, particle comminution and outflow). The use of intrinsic degradation characteristics as model inputs may be perceived as making modeling efforts partially self-fulfilling instead of having the mechanisms explain the physical and biological processes that underlie variation in substrate degradation rate (Baldwin, 1995). In a later version of the model, intrinsic *in situ* degradation characteristics were introduced to improve the model’s capacity to explain observed variation in feed degradation (Hanigan et al., 2013). Hence, a large part of the observed variation in substrate degradation remains dependent on degradation characteristics that relate to the chemical and physical properties of feeds and are truly intrinsic, independent of the mechanisms or calculation rules represented in both static and dynamic rumen models. Therefore, they are required as inputs to virtually all rumen models.

Apart from substrate degradation characteristics, further aspects and details about factors influencing microbial activity are included in rumen models, depending on the specific modeling aims. With the aim of predicting microbial protein supply, Dijkstra et al. (1998) compared various conceptual approaches and concluded that it is highly relevant to represent the mechanisms that underlie the interactions between micro-organisms and substrates. Because a substantial, but variable, proportion of gross microbial protein synthesis is recycled within the rumen without flowing to the duodenum, the interactions between different functional classes of micro-organisms were included. These interactions include predation (of bacteria and protozoa) by protozoa and microbial death (lysis), competition for substrates in the rumen, and differentiation of substrate requirement for growth and maintenance, of susceptibility to rumen outflow and of sensitivity to rumen pH. Although the importance of these microbial interactions is evident and has been reviewed frequently (e.g., Dijkstra et al., 1998; Firkins et al., 2007), there are hardly any attempts to include them in model representations of rumen fermentation dynamics. For example, in the Cornell Net Carbohydrate and Protein System (CNCPS), protozoal predation of bacteria is accommodated by a fixed 20% decrease in maximum growth yield of bacteria, independent of dietary or rumen characteristics (Russell et al., 1992). An exception to this is the model of Dijkstra (1994) which adopts a multispecies approach by representing the different functionalities of amylolytic bacteria, fibrolytic bacteria and protozoa, including their interactions. Such an approach is fundamentally different from the approaches used in the aforementioned static models, which may represent multiple microbial classes but lack a representation of dynamic interactions, making the final modeling outcome for the whole rumen the arithmetic summation of the outcomes of the representations for the individual functional classes. Recently, other modeling efforts have been undertaken to improve the representation of interactions between functional classes of micro-organisms (review Tedeschi et al., 2014). For example, a new version of the rumen submodel in CNCPS was constructed that includes more detailed aspects of growth and activity of Holotrichs and Entodiniomorphid protozoa (Higgs, 2014). Also, the AUSBEEF model is developed further based on the earlier work of Nagorcka et al. (2000), based again on Dijkstra (1994).

#### Dynamics of Rumen Metabolism

The importance of dynamic representation of the interactions between substrate and micro-organisms becomes apparent when investigating the effect of inflow rate of a single starch source on its ruminal degradation *in vivo*. Starch, as an example, is more illustrative in this respect compared to other substrates for the following reasons. In the rumen, almost all fiber is present in large particles, which become exposed to microbial degradation and rumen outflow after comminution and rumination, following a substantial delay, which is not or far less the case with starch (given processed starch sources are used). Fiber also has a substantial undegradable fraction which confounds the observed interaction between degradable fiber and fibrolytic micro-organisms, again in contrast to starch which in principle is fully degradable. Sugars typically have too small a rumen pool size and too high a digestibility to serve as an illustrative example. Also, an extensive exchange of N with the rumen wall and intra-ruminal N recycling (protozoal activity) complicates the case of N. The benefit of adopting a dynamic approach to explain rumen starch digestion does not readily become apparent from *in vivo* observations because effects on rumen starch digestion are masked by variation in dry matter (DM) intake, origin of starch, starch content and rumen outflow rate among treatments. Larsen et al. (2009) evaluated four experiments in which the effects of starch source and processing were examined. The results do not indicate that rumen starch digestibility of various cereal starch sources was affected by the level of starch intake. The depicted data for ground cereal starch do suggest an increased starch digestibility with increase of starch intake, but different cereal starch sources are compared here, level of DM intake varied, and starch intakes were always in the higher range (>4.5 kg/d). Some rumen digestion trials allow a direct comparison between starch treatments because an (almost) identical starch source was used across treatments (e.g., Herrera-Saldana et al., 1990; Poore et al., 1993; Joy et al., 1997). These studies indicate increased apparent rumen digestibility of starch source with increased intake of that source. Under the assumption that these effects were not caused by differences in fractional outflow rate, they might be explained by a larger pool of amylolytic micro-organisms in the rumen, and a larger capacity to degrade starch being present. Dynamic rumen models (**Figure 1A**) accommodate such an interaction between starch substrate and amylolytic microbial activity. A static model (**Figure 1C**) or a dynamic model with constant coefficient values which can be solved analytically and can be reduced to a static model description (**Figure 1B**) *a priori* do not. Another aspect to take into account with modeling is the contribution of microbial synthesis of storage polysaccharides with measurements of rumen starch digestion. These storage polysaccharides are included in analysis of rumen starch outflow, hence lowering apparent rumen starch digestion. Larsen et al. (2009) deliver strong empirical support for this effect of microbial starch. Their evaluation indicated that observed apparent ruminal digestibly underestimates true ruminal digestibility at low starch intake, due to a relatively higher contribution of microbial sources to total duodenal starch flow compared to rumen escape feed starch.

**FIGURE 2:**
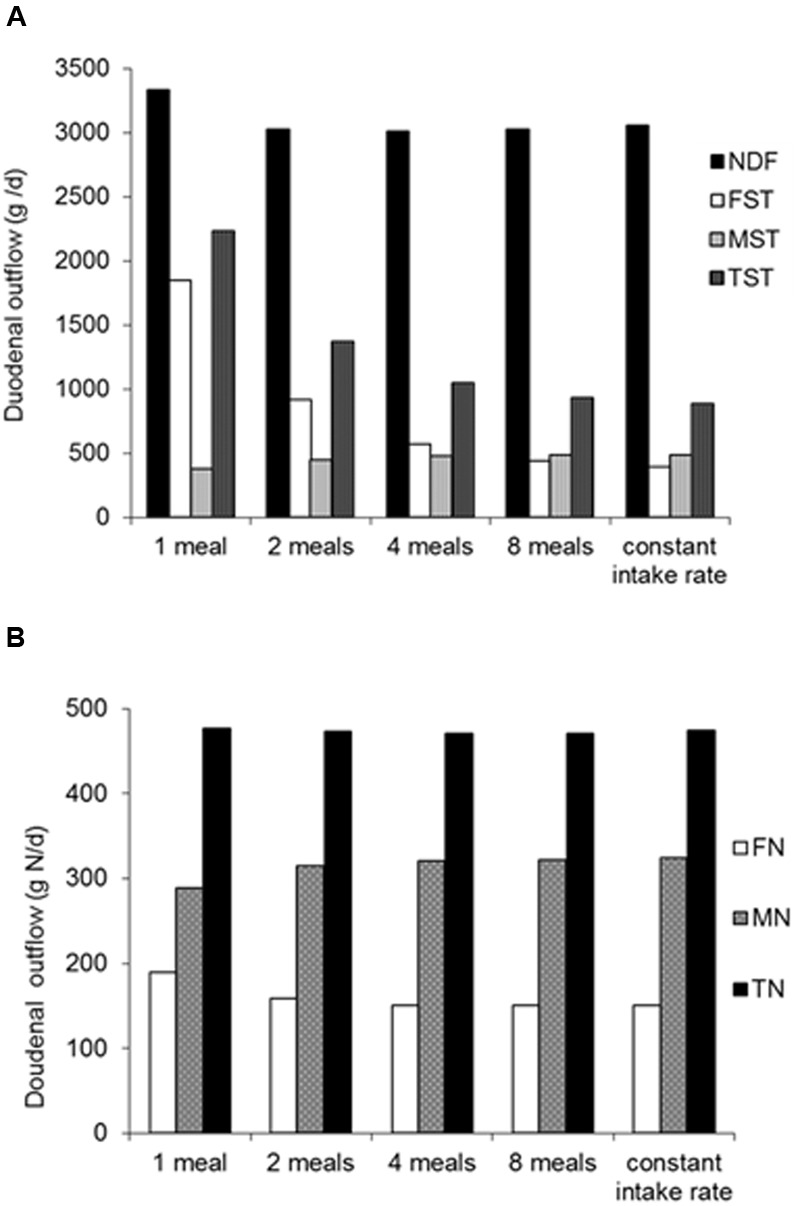
**Effect of feeding frequency on rumen digestion.** Simulations for different feeding frequencies were performed with identical diet composition (average Dutch dairy diet in 2004 with 13% grass herbage, 34% grass silage, 24% maize silage, 26% concentrates and 3% wet by-products in dietary DM). Further assumptions were a DM intake of 20 kg DM/d, a fractional passage rate of 1.0/d for particulate matter and 3.0/d for fluid, a mean rumen pH of 6.0 and a proportion of 0.35 of protozoa in total amylolytic microbial mass. An adapted version of the rumen model of Dijkstra et al. (1992) was used, including a representation of distinct meals, a mechanism for particle dynamics (distinction between large and small particles; a 0.4 proportion of small particles in feed was assumed and a fractional rate of comminution of large to small particles of 3.5/d). The contribution of individual meals to *in situ* degradation characteristics of rumen substrate pools was continuously monitored during the simulation runs. **(A)** Simulated duodenal outflow (g/d) of fiber (NDF), feed starch (FST), microbial starch (MST; engulfed starch granula as well as microbial polysaccharide synthesized), total starch (TST). **(B)** Simulated duodenal flow (g N/d) of feed N (FN), microbial N (MN) and total N (TN).

Observations on the effect of feeding frequency on rumen fermentation are a further illustration of the dynamics invoked. When a representation of meal intake pattern and a mechanism for particle dynamics are introduced into a dynamic rumen model (we use the rumen model of Dijkstra et al. (1992), for the sake of demonstration), a significant increase in rumen starch digestion and decrease in duodenal starch flow is simulated (**Figure 2A**) when moving from single to twice daily feeding. Lower starch degradation when feeding once daily corresponds to the results of Froetschel and Amos (1991), who showed an increase in fractional turnover rate of rumen starch of 75% with increase in feeding frequency from once to twelve times daily with a ground corn and sorghum silage diet, whereas the increase was limited to 24 and 1% for rumen DM and NDF, respectively. Le Liboux and Peyraud (1999) tested twice versus six times daily feeding of identical diets of (dehydrated) whole-crop maize and either ground or chopped alfalfa. More starch was ingested with six times feeding due to a higher DM intake, which resulted in a trend for increased starch digestion. Similar to these observations, the model simulations indicate a moderate effect of such an increase in feeding frequency from twice to four times daily on rumen starch digestion (**Figure 2A**). Le Liboux and Peyraud (1999) also observed that rumen digestion of digestible NDF increased with increase in feeding frequency, probably due to less fluctuation in rumen pH and improved rumen fibrolytic activity. This will not have played a role in starch digestion as it tends to be rather unaffected by pH. The present model simulations showed hardly any effect of feeding frequency on rumen digestion of NDF (**Figure 2A**) and N (**Figure 2B**), as model assumptions did not account for changes in rumen pH and fractional passage rate.

**FIGURE 3:**
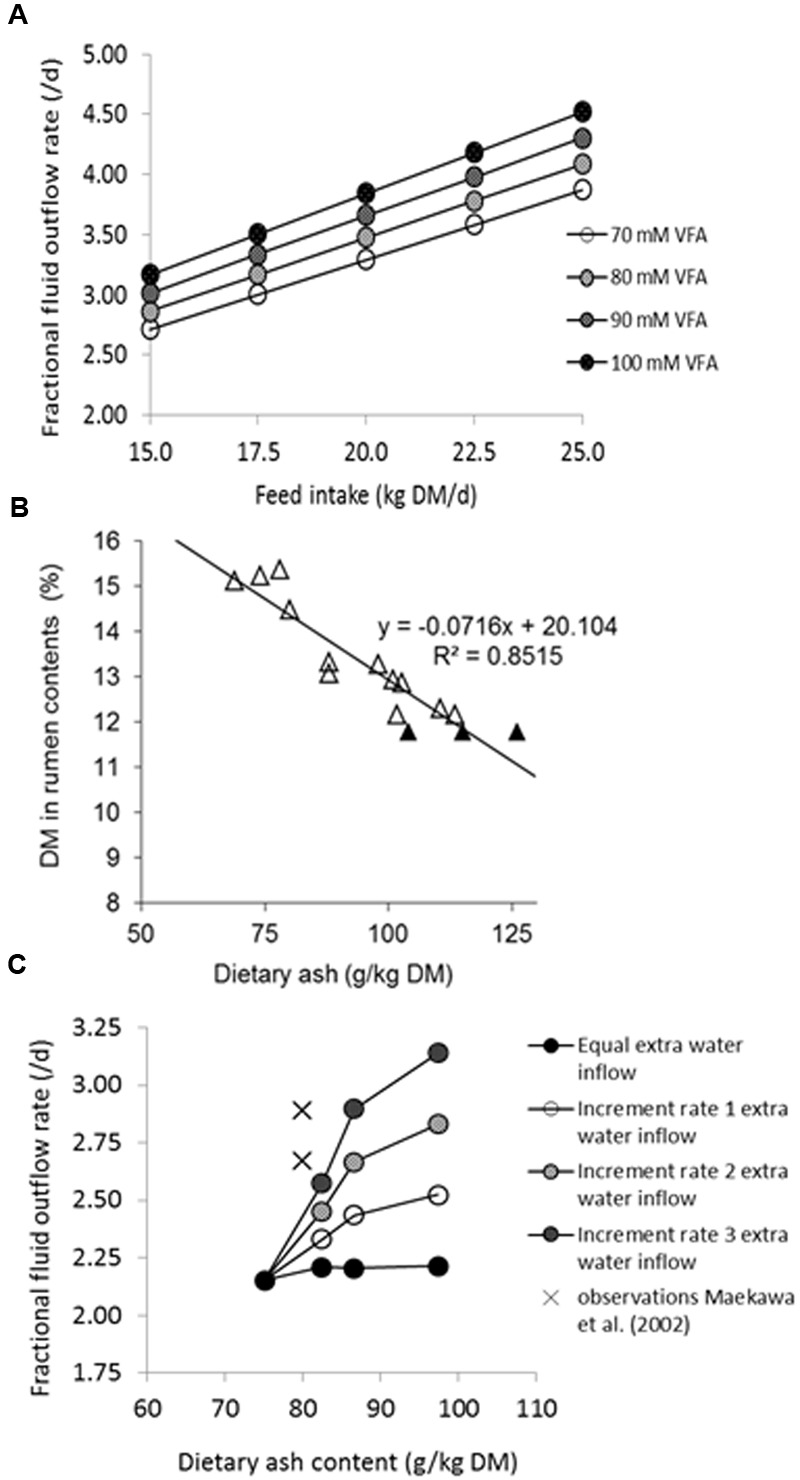
**Effects of dietary ash on rumen fluid dynamics. (A)** Simulated effect of DM intake (kg/d) on the fractional rate of fluid outflow as simulated by the model of Mills et al. (2001), increased by 5.6% per 10 mM increase in rumen VFA concentration as established for the sheep rumen by López et al. (2003) in the range of 50–80 mM VFA, and presumed to be applicable to a physiologically more realistic range for the dairy cow rumen of 70–100 mM VFA. **(B)** The effect of dietary ash content (g/kg DM) on proportion of DM (% of total weight) in whole rumen contents [open symbols for data from De Visser et al., 1992, 1993; Chilibroste et al., 2001; Bruinenberg et al., 2004; closed symbols independent data (average, and average ± standard deviation) from Reynolds et al., 2004]. **(C)** Effect of observed water intake with increase of dietary ash (salt) content (g/kg DM; according to Spek et al., 2012), adopting a rumen water volume based on the relationship depicted in **(B)**, and four scenarios of extra water inflow (an equal extra water inflow of 100 L/d, or an incremental rate 1, 2, or 3 of extra water intake of 1.4, 2.7, or 4.1 L/d per %DM increase in dietary ash content, respectively) on calculated fractional outflow rate of rumen fluid. Observed values for primiparous and multiparous lactating cows derived from Maekawa et al. (2002) are indicated by crosses.

The stimulatory effect of feeding frequency on rumen digestibility observed by Le Liboux and Peyraud (1999) contrasted with a consistently negative quantitative effect observed for digestibility of the feed determined at the level of fecal excretion (i.e., fecal digestibility). This may be explained by less substrate inflow and hence a smaller microbial population in the large intestine, resulting in a reduction in the contribution of the large intestine to OM and NDF digestion which was larger in size than the increased contribution by the rumen. Unless caused by bias introduced with measurement methodology, such effects can also be seen as illustrative of the previous discussion on the dynamics involved with interactions between substrate and micro-organisms in the rumen. This warrants fermentation in the large intestine to also be represented in a dynamic model. Therefore, we introduced a dependency of the fractional outflow rate of digesta on VFA concentration, assuming an increase of 10 mM is associated with a 5.6% increase of fractional outflow rate up to a maximum of 80 mM (taken from the results of López et al., 2003), into the dynamic model of the large intestine described by Mills et al. (2001). The consequence of this model adaptation (**Figure 3A**) was a marked decline in predicted fibrolytic activity with an increased inflow of rapidly fermentable carbohydrates into the large intestine. A similar response of a declined contribution of the large intestine to NDF digestion with an increased ileal starch flow has been reported in several studies (Knowlton et al., 1998; Yang and Beauchemin, 2005; Van Vuuren et al., 2010). Although the rather theoretical model simulations require confirmation, these *in vivo* results illustrate the dynamic aspects of how substrate availability and activity of amylolytic and fibrolytic micro-organisms impact NDF digestion beyond the rumen, when feeding large amounts of starch relatively resistant to rumen degradation. Also, the importance of addressing these dynamic aspects was highlighted in a recent modeling study, where Ellis et al. (2014) evaluated predictions of digestion and enteric methane (CH_4_) emissions in beef cattle against *in vivo* observations. For realistic predictions they first had to make changes to the rumen and large intestinal model of Mills et al. (2001), which was developed for lactating cows, to accommodate the specific enteric conditions observed in beef cattle fed low-roughage diets. The dynamics of digesta volume and the fractional outflow rates for the rumen and large intestine had to be modified, as well as the fractional outflow rate of rumen protozoa, and large intestinal fibrolytic activity, to achieve realistic simulations of fibrolytic activity in both the rumen and large intestine. Gregorini et al. (2015) recently revisited representation of digesta outflow in the mechanistic model of Baldwin (1995), addressing effects of osmolality of rumen fluid (calculated from solutes including VFA) on rumen fluid dynamics.

#### Influencing Factors

Other factors, as well as the characteristics of substrates and rumen micro-organisms, have a profound impact on rumen function and can cause seemingly contrary results for starch degradation to those discussed earlier. For example, the results of Sutton and Oldham (reported by Mills et al., 1999) fail to confirm the positive effect of higher intake of a single source of barley or maize starch on rumen starch digestion. In this case, rumen starch digestion was reduced rather than increased for maize and remained the same for barley. This outcome may be related to the low proportion of roughage in dietary DM with the high starch level compared to the low level, as studies mentioned in the previous section always tested diets where roughage accounted for >35% of dietary DM. The discrepancy illustrates that other mechanisms must override the effect of an increased starch inflow on rumen starch digestion, with turnover or outflow rate of rumen contents as the most likely candidate. The same was suggested by the findings of Ellis et al. (2014) for representing the digestion of low-roughage diets in beef cattle. Also in contrast, Kreikemeier et al. (1990) observed an increased *in situ* fractional rate of starch degradation with increased roughage in a wheat-based finishing steer diet, in spite of a reduced starch intake, a lower rumen starch pool, and a higher fractional outflow rate of labeled starch. Given that bacterial and protozoal numbers were not affected by increased dietary roughage, Kreikemeier et al. (1990) explained this result by an increased fractional liquid passage rate that improved the efficiency of microbial growth.

The need for an integrative approach to understand these phenomena of rumen function becomes more apparent when attempting to represent the aforementioned dynamics of substrate degradation and microbial metabolism, and the modulating effect of rumen conditions. For example, fiber degradation rate is affected not only by the fractional outflow rate of particulate matter but also by the acidity of rumen contents with pH under 6.3. The influences of rumen fluid and particle dynamics, of rumen fluid volume and rumen fill, of rumen fluid pH and of VFA absorption are represented in essence in every dynamic rumen model, albeit in a very different manner. Such differences are due to the different assumptions made or due to the different empirical bases used to parameterize the dynamic (Baldwin et al., 1987; Dijkstra et al., 1992) or static models which cover these aspects (Danfaer et al., 2006; Tylutki et al., 2008; Volden, 2011). Volume and fractional outflow rate of rumen fluid have a major effect on rumen concentrations of substrates and micro-organisms (Argyle and Baldwin, 1988). Next to saliva production, several additional aspects are important to quantify dietary impact on rumen fluid dynamics which will be demonstrated here using some theoretical calculations. **Figure 3A** illustrates the hypothetical effect of VFA accumulation in rumen fluid on net fluid exchange in the direction from blood to the rumen, driven by osmotic pressure, as demonstrated in sheep by López et al. (2003). **Figure 3B** illustrates the effect of dietary ash content on rumen fluid volume (derived from *in vivo* observations of DM% in total rumen content). This higher rumen fluid volume is accompanied by extra water intake, affecting fluid dynamics and concentrations of substrate and substrate fermenting micro-organisms in the rumen ecosystem. **Figure 3C** illustrates the impact of several scenarios for incremental extra water intake with increased dietary ash content on calculated fractional outflow rate of rumen fluid. A comparison with observations by Maekawa et al. (2002) seems to indicate that the highest incremental rate of extra water intake is realistic for lactating cows. Aspects that have received less attention in efforts to model the rumen are rumen fill, water holding capacity, fluid osmotic value, viscosity of rumen contents, rumen retention of fluid and its consequences for the different functional classes of feed particles and micro-organisms, and buffering capacity of rumen contents. These aspects significantly impact fractional outflow rate and rumen pH, both main drivers of rumen function in all rumen models (dynamic as well as static). They have received most attention in the modeling work of Argyle and Baldwin (1988; overview by Baldwin, 1995) which was recently revisited by Gregorini et al. (2015). Next to scientific purpose for proposing a mechanism and increase understanding, the integration of this type of aspect in rumen models widens the domain of their use. The alternative of not integrating them would be to reparameterize rumen models by a fully empirical approach to allow them accommodate the various rumen conditions or nutritional strategies.

### Classes of Microbial Function

**FIGURE 4:**
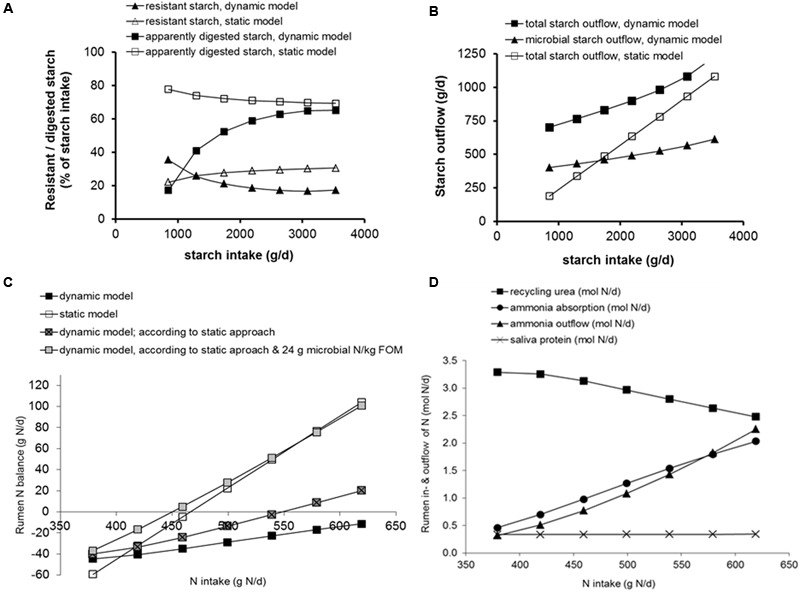
**Effect of substitution of maize silage for grass silage (up to 70%) in a diet containing 80% roughages and 20% concentrates on a DM basis, assuming a DM intake of 20 kg DM/d, simulated by the model of Dijkstra et al. (1992) as well as by a current protein evaluation systems (Van Duinkerken et al., 2011), as examples of a dynamic and a static rumen model, respectively (results derived from Bannink et al., 2006a). (A)** Resistant or apparently digested starch as % of starch intake simulated with the dynamic and the static rumen model. **(B)** Rumen outflow of microbial starch (microbial polysaccharides synthesized, and starch engulfed by micro-organisms) and total starch simulated with the dynamic and the static rumen model. **(C)** Rumen nitrogen balance simulated with the dynamic and the static rumen model, with the dynamic rumen model for the concept of rumen protein balance represented in the static model, and with the dynamic rumen model for the concept of rumen protein balance represented in the static model with a constant 24 g microbial N synthesized per kg rumen fermented OM (FOM) as assumed in the static model. **(D)** Simulation results for N flows to and from the rumen compartment with the dynamic rumen model (urea recycling, ammonia outflow, ammonia absorption, and saliva protein inflow). These flows as such are not represented in the static model.

Reasons to distinguish functional classes of micro-organisms in rumen models are differences in the type of substrate fermented (type of carbohydrate in particular), the particular rumen niche they occupy with its own physical-chemical characteristics, e.g., retention time and acidity, and the specific role they exert that is related to the modeling aim; e.g., bacterial predation by protozoa and storage of polysaccharides (Dijkstra et al., 1992; Dijkstra, 1994; **Figures 4A,B**) and rumen lactate metabolism (Mills et al., 2014). Mechanistic rumen models distinguish fibrolytic and amylolytic microbial activity because such a classification is well documented and in line with the distinct carbohydrate substrates for which intrinsic degradation characteristics are available. This is exclusively a distinction based on type of substrate and far less inspired by rumen-ecological arguments which would require representation of other types of variation. Such a representation may include variation in specific niches, diversifying the current distinction between particulate and fluid associated micro-organisms. It may include variation in specific rumen fermentation conditions, introducing intra-rumen compartmentation or gradients with respect to substrate availability and concentration of fermentation end-products. Or it may include variation in some unique role or metabolic state that rumen micro-organisms can achieve. Representing the latter may be the introduction into the model of metabolism of specific nutrients or metabolites or the identification of different metabolic states or responses of micro-organisms. These different states or responses might be due to a changed energy requirement for maintenance functions (Baldwin, 1995), or to regulatory mechanisms driven by changed availability of reduced co-factors (Van Lingen et al., 2016). With respect to representing the dynamics of inter-microbial relationships between various classes of micro-organisms, amongst the best documented and detailed models are the rumen protozoa metabolism model of Dijkstra (1994) and the rumen lactate metabolism model of Mills et al. (2014). Some other additions to rumen models can be considered as model parameterization rather than addition of a functional class of micro-organisms if they do not include representation of the dynamics of this functional class and its inter-relationships with other classes. For example, Nagorcka et al. (2000) introduced a consensus stoichiometry of VFA production derived from microbiological studies on individual bacteria genera and types of protozoa into the model of Dijkstra (1994), but no new dynamics were introduced. They used general microbiological data and not *in vivo* observations to describe the profile of rumen VFA in relation to substrates degraded. Whether the extra detail introduced by such a consensus outweighs the benefit of an empirical approach based on *in vivo* measurements in the target animal (Bannink et al., 2006b; Ghimire et al., 2014) remains unclear. Other aspects that have been incorporated in the various rumen models reported in literature include effective fiber, peptides, distribution of particles, fats and fatty acids, and sulfate (review Tedeschi et al., 2014), which have their own merit when aiming to explain rumen response to related factors.

A reason to introduce more detail on microbial activity into future rumen models, instead of representing a single type of micro-organism, might be the different niches that different species or classes of micro-organisms occupy. Another reason might be the differences in metabolic characteristics and growth capacity of micro-organisms, instead of making generic assumptions about amylolytic bacteria, fibrolytic bacteria and protozoa. Also differences in the sensitivity of microbial activity to conditions in the rumen environment instead treating it as fully independent, might be a reason. Currently, we are unaware of modeling efforts or new approaches to further distinguish microbial classes in order to increase understanding of whole rumen function. Although new molecular techniques in principle enable measurement up to the level of detail of the individual species or genus, such information would be particularly useful if it could be related to the functional aspects of rumen fermentation already represented in whole rumen models [i.e., rates of substrate degradation, microbial growth rates, formation rates of end-products of fermentation, and rumen fermentation conditions (fractional outflow rates, pH, and VFA)]. For example, effects of subclinical acidosis on the rumen microbiome have been observed by Mao et al. (2013). Also Petri et al. (2013) were able to establish clear relationships between the observed microbial profile and dietary treatment and acidotic challenge. Others, however, could not establish a relationship between observed rumen fermentation parameters (including severity of acidosis) and shifts in the rumen microbiome, and a large variation existed between individual animals and measurement periods (Mohammed et al., 2012). Having *in vivo* rumen measurements or data on microbial physiology alongside data from modern molecular techniques and the wealth of sequence-based information seems pivotal in relating such data to whole rumen function and using them to make progress in rumen modeling. A completely statistical approach can be adopted to relate this level of detailed information directly to observed rumen function without representing the underlying mechanisms. Although useful to mark the consequences of different rumen states, e.g., by deriving microbiome profiles marking a specific rumen (or even animal) state (Chaucheyras-Durand and Ossa, 2014), the challenge remains to go beyond a fully empirical approach (Bannink et al., 2011; Weimer, 2015) and use this information to contribute to concepts that can be applied to the rumen modeling approaches discussed herein (and vice versa).

Details of a specific functional microbial class can be added to a model to allow prediction of the associated functional response. For example, Mills et al. (2014) integrated a representation of lactate metabolism within the rumen model of Dijkstra (1994) by separating a single class of amylolytic bacteria into lactate-utilizing and lactate-producing bacteria. Except under strongly acidic conditions where lactobacilli become prominent, *Streptococcus bovis* is a major contributor to lactate production in the rumen (Nagaraja and Titgemeyer, 2007). In the model, growth parameters for lactate-utilizing bacteria were based on those reported for *Megasphaera elsdenii*, the major lactate utilizer in the rumen. The capability of the model to simulate peaks in rumen lactate post-feeding can be considered encouraging. When combined with an improved representation of rumen acid-base chemistry and sensitivity of the microbial classes to rumen pH, the model should become a useful tool for increasing our understanding of how nutritional factors (non-structural carbohydrate feeding, feed intake patterns) lead to lactate and VFA accumulation in the rumen, which can be highly detrimental to rumen function. Due to saliva production and its buffering capacity in the rumen, prediction of pH from rumen VFA concentration in current empirical relationships is inaccurate. In the case where saliva is absent, prediction of pH from VFA concentration is far more accurate, as shown for pH of fresh cow feces by Dijkstra et al. (2012). Therefore, there is much scope to model rumen acid-base chemistry and improve pH prediction under a wide range of rumen conditions (Imamidoost and Cant, 2005).

In addition to microbial classes in the rumen ecosystem, further distinction of specific microbial metabolic functions can also be relevant. The models of Dijkstra et al. (1992) and Dijkstra (1994) attempt to capture the variation in microbial composition as a key aspect of microbial metabolism. Amylolytic bacteria and protozoa are able to store polysaccharides. This functionality is important when predicting sugar or starch outflow to the intestine as glucogenic precursors. It may also be an important buffering mechanism acting against sudden, large loads of rapidly fermentable carbohydrate and temporal rumen acidification, as the carbohydrates stored will not generate rumen VFA during these moments. Later on, part of these storage carbohydrates re-enter fermentation with lysis of micro-organisms and contribute to VFA production, and part flows out of the rumen with microbial matter (Dijkstra et al., 1992; Dijkstra, 1994). In **Figures 4A–D**, outcomes of such microbial composition dynamics are illustrated and compared to outcomes from static modeling approaches for the dietary substitution of maize silage for grass silage. With the static approach, a small decline in percentage of starch digested (and increase of starch resisting digestion) is predicted with an increasing proportion of maize silage in the diet (**Figure 4A**). This outcome is the combined result of an increasing proportion of maize silage starch at the expense of concentrate starch in the diet, and a lower rumen degradation rate for maize silage compared to concentrate starch. With the dynamic approach a different trend for starch digestion was predicted due to representation of microbial storage of carbohydrates. Microbial storage of polysaccharides contributes to measured rumen starch outflow and as a result, at a very low starch intake, the percentage of apparently digested starch remains much lower and the percentage of resistant starch higher compared to outcomes with the static approach. Because the represented microbial capacity of storage of polysaccharides is limited in size, with increasing starch intake the impact of microbial polysaccharides on predicted results declines and predictions of apparently digested starch approach those obtained with the static approach. Due to the contribution of microbial polysaccharide storage to resistant starch (as measured in rumen digestion trials), predicted daily starch outflow remains higher with the dynamic compared to the static approach, even at high levels of starch intake (**Figure 4B**). Such contrasting results from both modeling approaches can be evaluated against observed phenomena on rumen starch digestion. Although any expected variation in (the biosynthesis of) components of microbial matter would have major impact on the quantification of microbial synthesis and predicted rumen metabolism, no other modeling examples other than for microbial polysaccharides are known that take this aspect into account.

A further example of adding specific functionality to a rumen model is the work on modeling rumen fat metabolism by Dijkstra et al. (2000) and Moate et al. (2004). A comparison of both studies illustrates how different modeling aims lead to different modeling approaches, although they both strive to represent the key details of rumen fat metabolism. The former model is dynamic with a simplified representation of fat source, by only distinguishing saturated from unsaturated fat and hydrolyzed from non-hydrolyzed fatty acids. It does, however, include a representation of how fatty acid concentrations in the rumen interact with microbial activity and fiber digestion. The latter model is static and aims to represent details of the type and fate of dietary fatty acids. However, it excludes a representation of interactions with microbial activity and substrate degradation. Such modeling work is of considerable interest in studying the effects of dietary fat supplementation on milk fat composition and rumen fatty acid metabolism. With current interest in using milk fatty acid profiles as a proxy for enteric CH_4_ emissions (e.g., Van Lingen et al., 2014), details of rumen fat metabolism seem an essential link in being able to interpret such profiles. Accounting for the modulating effect of fat supplementation on rumen microbial activity and VFA production requires the prediction of rumen fatty acid concentrations, favoring a dynamic modeling approach. A combination of both approaches seems most promising in predicting all relevant effects (microbial metabolism, methanogenesis, fatty acid outflow).

### Non-microbial End-Products

In addition to the representation of microbial protein synthesis, reviewed by Dijkstra et al. (1998), and which may include representations of various functional microbial classes, further details can be added to explain the variation in rumen microbial metabolism. Such details would have to be integrated at the whole organ level to address the aims of rumen models, i.e., to generate predictions of microbial protein outflow to the intestine, and production of VFA, ammonia and CH_4_. The VFA as end-products of rumen fermentation are of particular importance because they deliver the majority of metabolizable energy to the ruminant. It is noted here that modeling of VFA production has been strongly hampered by much of this work being based on molar proportion of VFA found in rumen fluid instead of actually measured VFA production rates. The production of hydrogen (H_2_) is strongly associated with that of VFA, as it is used by methanogens to produce CH_4_. Recent attempts to further improve the description of microbial metabolism and predict the consequences for VFA and CH_4_ production are discussed here.

#### Microbial Metabolism

Hydrogen is an important metabolite in the fermentation of glucose to VFA in the rumen (Baldwin and Allison, 1983; Hegarty and Gerdes, 1999). Levels of H_2_ in the rumen depend on the amount of substrate fermented and type of VFA produced (mainly acetate, propionate and butyrate). Glycolysis, the primary pathway of glucose fermentation by rumen microbes, yields two equivalents of pyruvate and reduces two equivalents of NAD^+^ to NADH. This NADH must be oxidized back to NAD^+^ to maintain glycolysis. For thermodynamic reasons, this oxidation proceeds spontaneously at low concentrations of H_2_ and is decreased or even ceases at higher concentrations of H_2_. In other words, the H_2_ concentration in the anaerobic environment dictates the redox state of the cofactor (Zhang et al., 2013). Rumen methanogens may increase CH_4_ production when the H_2_ concentration is increased to utilize the accumulated H_2_ and, as a consequence, favor the re-oxidation of the reduced cofactor in bacterial cells. Based on the H_2_ emission profile shown in Rooke et al. (2014), this condition may occur directly after a meal, in particular after one containing a large proportion of rapidly degradable carbohydrates. Various microbial species are still able to oxidize NADH by reducing intracellular metabolites to ethanol, lactate or succinate (Stams and Plugge, 2009). The formation of lactate and succinate, being precursors of propionate, explains why elevated levels of propionate are associated with increased levels of H_2_ (Janssen, 2010; Van Lingen et al., 2016).

Janssen (2010) and Ungerfeld (2013) evaluated the thermodynamic effect of H_2_ concentration on various fermentation pathways in terms of Gibbs energy change (ΔG). Negative values of ΔG indicate a reaction will proceed in the forward direction, positive values indicate the reverse direction, and ΔG = 0 indicates equilibrium between the forward and reverse reactions. Janssen (2010) showed the ΔG of several fermentation pathways becomes less negative at increasing concentrations of H_2_ and argued that an increase in H_2_ concentration shifts fermentation away from H_2_ releasing pathways, resulting in increased propionate production. Ungerfeld (2013) showed the ΔG of the acetate to propionate conversion to become more negative at increased levels of H_2_. These thermodynamic investigations determine the energetic favorability of fermentation pathways at different H_2_ partial pressures in the rumen. However, ΔG is not a direct measure of the rate at which a reaction takes place, nor does it provide a direct measure of the extent to which changed rumen conditions affect VFA production rates. A further aspect that deserves attention is microbial biosynthesis, and modeling rumen microbial metabolism under various conditions would benefit from investigations into energetic costs of various microbial (biosynthetic) functions, but it is not a focus of current rumen modeling work. Some modeling work has been undertaken to improve the accuracy of the prediction of rumen VFA profiles based on these thermodynamic principles.

Initially by Ungerfeld and Kohn (2006), and later by Ghimire et al. (2014), interconversion rates of VFA were predicted by combining general laws of reaction kinetics with reaction quotients of chemical equilibria. These approaches go beyond previous ones with a strictly empirical description derived from rumen observations (to be discussed in the next section). However, these new approaches might still not be fully consistent with both reaction kinetics and thermodynamic control of reaction rates. Thermodynamic control on a particular reaction pathway is assessed from equations that have been derived from kinetic rate laws, with stoichiometric coefficients used as exponent values of reactant concentrations in these equations. However, physical chemistry text books such as that of Atkins and de Paula (2006) indicate that experimentally determined exponent values instead of stoichiometric ones have to be used for reactant concentration in kinetic rate laws; thermodynamic control on reactions might be realistically assessed by correcting the kinetic rate laws for thermodynamic effects (Van Lingen et al., 2016).

Ghimire et al. (2014) fitted rate constants for VFA interconversion from *in vivo* observations of rumen VFA interconversion with control treatments that were expected not to affect thermodynamic control (i.e., a roughage diet and saline infusion), and assumed a constant partial pressure of H_2_. They subsequently applied their fitted rate constants to predict VFA production for the other treatments which were expected to have a different thermodynamic control (i.e., a low roughage diet and intra-ruminal propionate infusion), and concluded that the model did not perform well in predicting ruminal VFA production rates due to lack of data on thermodynamic control factors other than pH and rumen VFA concentrations. Also, for an evaluation of thermodynamic control of rumen fermentation pathways, H_2_ partial pressure is best varied, as opposed of being kept constant. Prediction inaccuracy may also result from some limitations in their quantitative approach. Bannink et al. (2006b), who estimated coefficients for VFA molar proportions using a fully empirical approach, recommended inclusion of the dynamics of cofactor redox state as a controlling factor of VFA production rate and VFA molar proportions (in non-steady-state conditions). Mostly NAD^+^/NADH is considered, but for a more detailed discussion on this presumption the reader is referred to Van Lingen et al. (2016).

Offner and Sauvant (2006) introduced the dynamics of reduced cofactors in their model to predict end products of rumen fermentation, adopting a similar modeling approach as Ghimire et al. (2014). However, they chose their rate constants arbitrarily which might have caused unrealistically high (as high as 15) acetate to propionate ratios at certain moments when simulating the transient diurnal response. Better representation of thermodynamic controlled factors should improve the prediction of rumen VFA profiles by future mechanistic rumen models. This also holds for modeling CH_4_ production, considering VFA production and associated H_2_ are the main drivers for methanogens. However, this type of modeling work has rarely been reported. Also no examples are known of rumen models that describe the variation in microbial composition and biosynthetic functions (and consequences for metabolism; Hegarty and Gerdes, 1999) in response to rumen fermentation conditions, and its consequences for the activity of specific types or classes of rumen micro-organisms. Recently, Vetharaniam et al. (2015) introduced a representation of the dynamics of H_2_ and methanogens (represented as a constant fraction of rumen microbial pool size) to facilitate future consideration of thermodynamic control of CH_4_ production in their rumen model.

#### Fully Empirical Approaches

Since the early work of Koong et al. (1975) there have been numerous studies to derive the stoichiometry of rumen VFA production from rumen observations on substrate digestion and molar VFA proportions using empirical approaches (Murphy et al., 1982; Bannink et al., 2006b; Sveinbjörnsson et al., 2006; Nozière et al., 2011). Instead of using rumen VFA observations, Nagorcka et al. (2000) adopted a different approach by using microbiological data on VFA formed, for the first time separating VFA formed by bacteria (comparable to the other studies) from VFA with a relatively high amount of butyrate formed by protozoa. Nagorcka et al. (2000) reported an improved prediction of rumen VFA in lactating cows, heifers and growing cattle, compared to predictions obtained with the stoichiometry of Murphy et al. (1982) which was derived from steer observations. This outcome might partly be due to the stoichiometry of Murphy et al. (1982) being less applicable to the evaluation data set used by Nagorcka et al. (2000) due to differences in level of DM intake, diets and rumen fermentation conditions. The stoichiometry derived by Bannink et al. (2006b, 2008) was based strictly on lactating cow data resulting in VFA predictions that were shown by Morvay et al. (2011) to deviate substantially from those obtained using the stoichiometry of Murphy et al. (1982), and to deliver an improved prediction when evaluated against lactating cow data. This particular study evaluated the stoichiometries of Murphy et al. (1982), Argyle and Baldwin (1988), Friggens et al. (1998), Bannink et al. (2006b, 2008) and Sveinbjörnsson et al. (2006) against an independent data set for lactating cows. Predictive power for propionate and butyrate differed strongly among the alternative representations of VFA stoichiometry, and linear relationships between rumen pH and VFA production did not appear to improve prediction of VFA profile. Another evaluation study of enteric CH_4_ in beef cattle by Ellis et al. (2014) confirmed that inclusion of linear pH-dependency in VFA stoichiometry worsened CH_4_ predictions (reflecting VFA profile) whereas non-linear dependency did not. The calculations on the VFA stoichiometry of Nozière et al. (2011) were not available to Morvay et al. (2011), but were used in a follow-up evaluation study on lactating cow data by Alemu et al. (2011). This study showed that both the stoichiometries of Bannink et al. (2006b) and Nozière et al. (2011) outperformed those of Murphy et al. (1982) and Sveinbjörnsson et al. (2006) in predicting VFA molar proportions. The stoichiometry of Nagorcka et al. (2000) was recently evaluated by Gregorini et al. (2013) following enhanced representation of the digestive elements in the model of Baldwin et al. (1987) by Hanigan et al. (2013). Prediction of CH_4_ emissions did not improve however, which probably reflects VFA prediction did not improve either.

When additives known to modify the VFA profile of the rumen are included in the diet, the limitations of empirical approaches in predicting VFA (i.e., their lack of relation to microbial metabolism) become even more evident. As an example, with monensin there is typically a reduction in ruminal gram-positive bacteria population size and activity, and this shift in bacterial population results in an increase in propionate production. Ellis et al. (2012) modeled the change in VFA profile with monensin dose in feedlot beef cattle and showed improved VFA predictions with this modified VFA stoichiometry. Combining the equations derived by Ellis et al. (2012) to correct predicted VFA profile for the effect of monensin in beef cattle diets also improved CH_4_ predictions when using pH-independent, but not when using pH-dependent VFA stoichiometry. However, the authors noted that the extent of VFA profile change will differ between dairy and feedlot beef cattle, which might be due to differences in the typical basal diet and rumen microbial population.

As discussed in the previous section, there is renewed interest in modeling the thermodynamic aspects of variation in VFA production empirically (Ghimire et al., 2014). So far, this does not appear to have improved our understanding of the variation in VFA stoichiometry, and mean squared prediction errors across studies typically remain around 10, 15, and 20% of observed proportions of acetate, propionate and butyrate, respectively. A lower concentration or molar proportion of individual VFA results in a higher mean squared prediction error, as model simulations by Bannink et al. (2006b) demonstrate. Although unclear which approach (mechanistic or fully empirical) unravels the driving factors of change in rumen VFA profile best, more elementary approaches to modeling metabolic aspects of rumen micro-organisms and thermodynamic control of their fermentation pathways (including the dynamics of cofactor redox state; Mosey, 1983) are welcome. Rumen pH is a less useful explanatory variable as it is likely not closely associated enough with these driving factors. If pursuing a fully empirical approach (i.e., based on rumen observational data only), future efforts would be best focused on introducing the effects of the intensity at which micro-organisms ferment substrates (reflecting reduced co-factor dynamics), with the effect of amount of substrate fermented as the main driver.

### The Rumen Organ

Almost all rumen modeling efforts have been directed at representing the chemical and physical processes that take place in the lumen. However, lumen conditions in the host are closely regulated by exchange of water and solutes, rumen motility and outflow, and rumination (Baldwin, 1995). Thereby the ruminant host has a profound impact on rumen microbial activity. Fully integrated models of rumen metabolism that include the functions of organ tissues are currently lacking. Two aspects in particular deserve to be discussed because of their importance in regulating rumen fermentation conditions: urea-N recycling and VFA absorption.

#### Urea-N Recycling

Conservation of N through urea-N recycling by the kidney and by the rumen wall is an important mechanism for ruminants to prevent N from limiting microbial growth with low N intake (Reynolds and Kristensen, 2008). This mechanism is represented differently in static and dynamic rumen models. Use of both types of model to evaluate the effect of N supply on rumen metabolism is demonstrated by comparing outcomes of the dynamic model of Dijkstra et al. (1992) with a static protein evaluation system currently applied in practice (Van Duinkerken et al., 2011). Simulations were performed for an exchange of maize silage for grass silage in a dairy cow diet, resulting in a continuous decline in N supply (**Figure 4**). The response of the dynamic model shows urea recycling to become saturated at the highest level of maize silage inclusion (i.e., 11.9% crude protein in dietary DM; **Figures 4C,D**), whereas the static model shows a linear decline in rumen degradable protein balance (reaching an ill-advised negative value at 14.4% crude protein in dietary DM; **Figure 4C**). *In vivo* measurements confirm the merit of adopting a more dynamic approach as it demonstrates the limiting effect of N supply on rumen fiber digestion at similar crude protein contents in dietary DM. A crude protein level of 11.5% in maize silage diets was tested by Spek et al. (2013) and a level of 10.3% in grass herbage diets was tested by Warner et al. (2015), which in both studies resulted in a rumen ammonia concentration below 2 mM which is considered to be well below critical levels for ensuring optimal microbial activity (Dijkstra et al., 1998). The static model calculated a rumen protein balance of around zero at this low protein level (Spek et al., 2013), hence falling within recommendations.

#### VFA Absorption (Adaptation)

**FIGURE 5:**
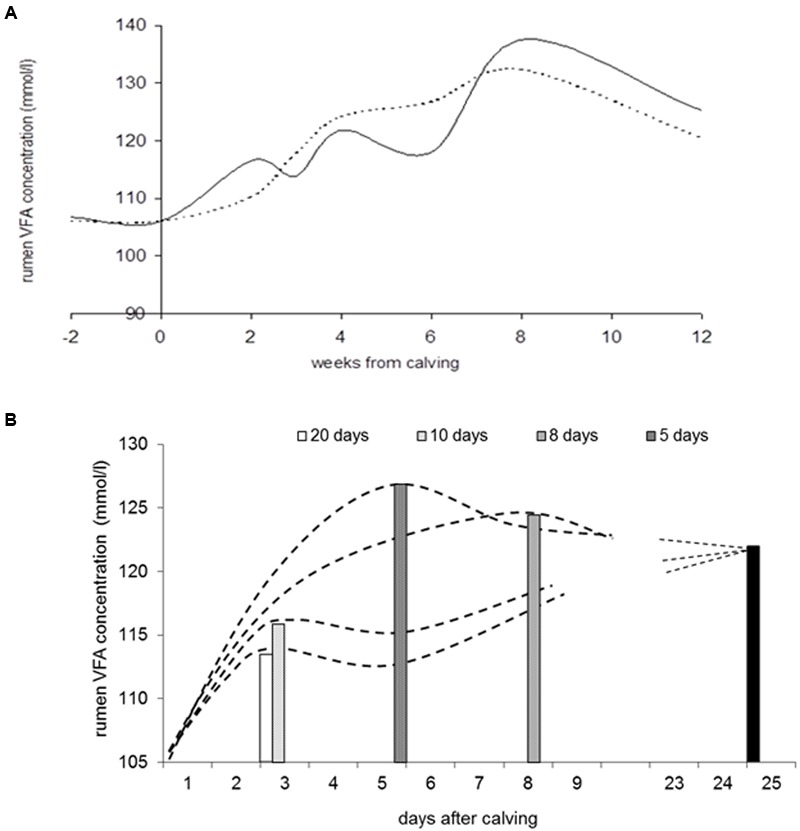
**Response in rumen VFA concentration with increment of concentrate allowance after calving (results reproduced from Bannink et al., 2012). (A)** Observed development in rumen VFA concentration with two rates of daily increment in concentrate allowance after calving to a maximum of 12 kg concentrate DM/d (in 10 days, solid curve; in 20 days, dashed curve). **(B)** Predicted development in rumen VFA concentration when increasing concentrate allowance to a maximum of 12 kg DM/d in 5, 7.5, 10, or 20 days after calving. The model describes rumen VFA absorption, growth of epithelial mass and surface area, epithelial VFA metabolism and VFA transport to portal blood in response to rumen VFA production. The model was calibrated using the trial for which results are depicted in **(A)**, including estimated rumen VFA production rate and observations on rumen epithelial tissue development. Bars indicate the moment maximum VFA concentration is achieved during the various strategies of increment of concentrate allowance; the black bar indicates steady-state VFA concentration achieved at 25 days after calving.

Maintaining rumen pH between narrow bounds is important for optimal fibrolytic activity. Regulatory mechanisms involve the buffering of rumen contents and removal of VFA (saliva secretion, VFA transport, VFA outflow). To our knowledge none of the published whole rumen models takes account of the changes in these buffering mechanisms with dietary and physiological transitions. *In vivo* studies with repeated measurements in transition cows reveal that the rumen wall adapts extremely quickly to changes in dietary regime through changes in morphology and histological characteristics (Bannink et al., 2012; Dieho et al., 2016). The faster of the two regimes for incrementing concentrate allowance after calving more than doubled the rate of VFA production as measured using the stable isotope technique, but the rumen wall epithelia appeared to adapt within 2–3 weeks. This adaptive response moderated the effect of the rapid increase in VFA load after calving to only modest changes in rumen VFA concentration and rumen pH (**Figure 5A**). Based on observed dynamics of rumen papillae size and changes in epithelial tissue, Bannink et al. (2008) proposed a model containing lumen, epithelial tissue and arterial blood compartments and described the rate of increase in rumen epithelial mass and surface area in response to the increased VFA load epithelia are exposed to after calving. Predicted VFA absorption followed observed phenomena (e.g., López et al., 2003) when assuming a far higher VFA transport rate at the serosal side (reflecting extensive epithelia protrusions and higher VFA exchange capacity) compared to the mucosal side of the epithelia. In support of this, Storm et al. (2012) demonstrated a profound effect of epithelial blood flow on rumen VFA absorption rate by modeling observations of rumen VFA absorption and epithelial blood flow. They also concluded that properties of rumen epithelial membranes, epithelial metabolism and epithelial blood flow should be represented in future models of ruminal VFA kinetics. Furthermore, assuming epithelial adaptation as modeled by Bannink et al. (2012), prevention of an accumulation of VFA during the increment of concentrate allowance after calving was predicted for a wide range of strategies (**Figure 5B**). This result seems to contrast with the risk of development of (subclinical) rumen acidosis during early lactation due to a rumen wall that was not yet fully adapted. It invites further *in vivo* testing of hypotheses on the implications of rumen adaptive capacity on rumen function.

### Engineering Rumen Metabolism

The ever increasing capacity to analyze detailed information on the rumen metabolome allows researchers to study rumen microbial metabolism in far more detail than ever before. Assignment of rumen function to rumen microbial genes is required to link the rumen microbiome to the nutritional and productive state of ruminants (Morgavi et al., 2013). Recently, Henderson et al. (2015) established a varying microbiome across a wide range of host species and dietary conditions worldwide, showing a more prominent effect of diet than of host. Metabolic interactions also appeared non-selective rather than specific, indicating a high functional redundancy and flexibility of the rumen microbial community at the level of detail that was examined (Henderson et al., 2015). There is a gap, however, between the information at the level of the rumen microbiome (i.e., microbial diversity, abundancy and gene expression) and the level of rumen microbial metabolism represented in whole rumen models (microbial population present, enzymatic degrading capacity, microbial growth characteristics) that needs to be bridged. Many studies lack measurement of rumen functionality and give the rumen microbiome or metabolome as an ordinal response to a pre-selected set of conditions or animal phenotypes. McCann et al. (2014) discussed the apparent absence of an association between residual feed intake of cattle and the rumen microbiome because it is either too subtle to discern, or the microbiome is biologically less significant in explaining genetic diversity in this phenotypic trait, or this trait varies with diet and feeding strategy. It may also be that the degree of detail by which the microbiome is characterized is still inadequate. Ross et al. (2012) concluded a method of untargeted massively parallel sequencing can be applied to characterize the relationship between rumen microbial community metagenome and economically or environmentally important traits (e.g., feed conversion efficiency and methane emission), but with the important constraint that rumen samples are taken at the same time and cows are fed the same diet. On top of this, standardization of the rumen sampling procedure is prerequisite as this strongly affects results. Yáñez-Ruiz et al. (2015) reviewed the development of the rumen in the context of early life development of rumen function determining host-microbiome specificity at the adult stage. They concluded the rumen needs to be understood in terms of the interplay between anatomical/functional and microbial aspects during early life. Despite these host-microbiome specificities and possibility of microbiome programming, the diet remains the most important driver of the rumen microbiome at the adult stage (McCann et al., 2014; Henderson et al., 2015).

Methodological aspects are also important for analysis of the relationship between rumen microbiome and rumen or animal function. Jami et al. (2014) reported the ratio of rumen Firmicutes to Bacteroidetes explains 51% of variation in milk fat yield by dairy cows on the same dietary treatment, and suggested the rumen microbiome modulates milk composition. However, it remains unclear whether other factors than the rumen microbiome might explain such observations just as well; milk fat content and DM intake explained only very small proportions of observed variation in milk fat yield (15 and 3%, respectively). Furthermore, it is noted that analysis of a rumen sample does not directly reflect whole rumen function in terms of gene expression, enzymatic capacity, microbial growth, VFA production or methanogenesis. A rumen sample can only deliver a measurement of concentration, expression or activity in that particular sample of rumen digesta. Even when the sample is taken from evacuated rumen contents, and is therefore representative of whole rumen contents, the sample will still not deliver an estimate of the total quantity, expression or activity present in the rumen as a whole. In this respect, there is a clear parallel with VFA measurements in rumen fluid samples not being representative of treatment effects or VFA production rate (Hall et al., 2015). In a recent review, Weimer (2015) concluded that advances in determining community composition and diversity have outpaced the ability to factor out the physiological and ecological roles of individual phylotypes that impact rumen function and animal performance. A high redundancy, strong resilience and host specificity of the rumen microbiome (Weimer, 2015) complicates the delineation of these precise roles, however, which remains a major challenge at present. To go beyond details of the variation in the rumen microbiome, observations of important drivers of whole rumen function and rumen functionality must be made (Bannink et al., 2011; McCann et al., 2014; Weimer, 2015). As discussed in the present paper, likely candidate rumen traits to observe are rumen volume, rumen outflow, the rumen microbiome, rumen substrate degradation, rumen VFA profiles and rumen fermentation conditions (e.g., pH). Insight into the interplay between these factors, to a sufficient depth or precision to allow them to improve upon current model parameterization, is required to account for these factors in rumen models and to make goal-directed use of variation in microbiome and in rumen functionality. It may, however, prove to be very difficult to gather all such details in conjunction with data on the rumen microbiome, and furthermore it puts stringent constraints on experimental designs.

## Conclusion

Use of mathematical models to advance our understanding of rumen metabolism requires appraisal of (i) the representation of metabolic factors that govern microbial metabolism and associated fermentation pathways, (ii) the diversity of functional classes or unique niches for micro-organisms needing to be distinguished, and (iii) the rumen functioning as an organ which includes the physiological mechanisms that regulate the rumen environment and exchanges with the ruminant host. Depending on the modeling aim, there is merit in representing the dynamic aspects of interactions between substrate degradation and microbial metabolism, and of interactions between the different functional classes of micro-organisms. The present review demonstrates how such dynamical aspects need to be represented in rumen models to explain observed phenomena in rumen metabolism. These phenomena include those observed with changes in feeding frequency and rumen fluid dynamics, changes in the profile of VFA produced, the regulatory mechanisms involved with N exchange between host and the rumen on low protein diets, and adaptation processes in rumen wall tissues to regulate rumen fermentation conditions.

Areas requiring more attention are the representation of (i) rumen fluid (and particle) dynamics (i.e., delineation of the processes contributing to fluid dynamics), (ii) the profile of VFA produced (i.e., representation of the impact of microbial metabolism on VFA profile), (iii) clearance of VFA from the rumen environment (i.e., representation of rumen VFA absorption capacity, including adaptive response of the rumen wall), (iv) the functional classes of micro-organisms needing to be distinguished. Emphasis should be placed on translating outcomes of rumen research in functional terms that can be integrated with efforts to model whole rumen function. These terms include substrate degradation rate, microbial metabolic costs for maintenance and synthetic purposes, composition of microbial mass, shifts in fermentation pathways, and interactions between classes of micro-organisms that one needs to distinguish from the perspective of their differing ecological roles in the rumen.

## Author Contributions

All authors listed, have made substantial, direct and intellectual contribution to the work, and approved it for publication.

## Conflict of Interest Statement

The authors declare that the research was conducted in the absence of any commercial or financial relationships that could be construed as a potential conflict of interest.
